# High School Students Are a Target Group for Fight against Self-Medication with Antimalarial Drugs: A Pilot Study in University of Kinshasa, Democratic Republic of Congo

**DOI:** 10.1155/2016/6438639

**Published:** 2016-06-02

**Authors:** Ramsès Kabongo Kamitalu, Michel Ntetani Aloni

**Affiliations:** ^1^Centre Hospitalier du Mont Amba, University of Kinshasa, Kinshasa, Democratic Republic of the Congo; ^2^Centre de Santé Universitaire, University of Kinshasa, Kinshasa, Democratic Republic of the Congo; ^3^Division of Hemato-Oncology and Nephrology, Department of Pediatrics, University Hospital of Kinshasa, School of Medicine, University of Kinshasa, Kinshasa, Democratic Republic of the Congo

## Abstract

*Aim*. To assess the self-medication against malaria infection in population of Congolese students in Kinshasa, Democratic Republic of Congo (DRC).* Methods*. A cross-sectional study was carried out in University of Kinshasa, Kinshasa, Democratic Republic of Congo. Medical records of all students with malaria admitted to Centre de Santé Universitaire of University of Kinshasa from January 1, 2008, to April 30, 2008, were reviewed retrospectively.* Results*. The median age of the patients was 25.4 years (range: from 18 to 36 years). The majority of them were male (67.9%). Artemisinin-based combination treatments (ACTs) was the most used self-prescribed antimalarial drugs. However, self-medication was associated with the ingestion of quinine in 19.9% of cases. No case of ingestion of artesunate/artemether in monotherapy was found. All the medicines taken were registered in DRC. In this series, self-prescribed antimalarial was very irrational in terms of dose and duration of treatment.* Conclusion*. This paper highlights self-medication by a group who should be aware of malaria treatment protocols. The level of self-prescribing quinine is relatively high among students and is disturbing for a molecule reserved for severe disease in Congolese health care policy in management of malaria.

## 1. Introduction

Malaria is a major public health problem with 300–500 million new infections each year and an estimated 584,000 deaths from malaria were reported worldwide, with most (90%) of the deaths occurring in Africa [1]. The decline of susceptibility of* Plasmodium falciparum* to chloroquine and sulfadoxine-pyrimethamine in many high-transmission areas resulted in the change of drug use policy in Africa [2]. In Democratic Republic of Congo (DRC), current recommendation therapy for severe malaria in DRC is artemisinin-based combination therapy (ACT); mainly, artesunate-amodiaquine has been recommended formally as a first-line antimalarial regimen since 2005 [3]. Quinine is reserved for severe or resistant forms of malaria. However, this policy is not controlled and the use of antimalarials without a prescription can have adverse effects on chemosensitivity in Kinshasa, the DRC [4]. Little information is available about self-medication in the DRC particularly in universities.

The aim of the present study was to assess the self-medication against malaria infection in population of Congolese students living in Kinshasa, DRC.

## 2. Population and Methods

This cross-sectional study was carried out in University of Kinshasa, Kinshasa, DRC. Medical records of all students with malaria admitted to Centre de Santé Universitaire (CSU) of University of Kinshasa from January 1, 2008, to April 30, 2008, were reviewed retrospectively. CSU is a primary level centre located in the city of University of Kinshasa, Kinshasa, DRC, and serving an estimated 30,000 students.

We used hospital-based data from the records. Students who experienced an episode of malaria and presented the following criteria, axillary temperature > 38.0°C with positive* Plasmodium falciparum* parasites in thick blood smears and absence of febrile conditions caused by diseases other than malaria, were included in this study. Students who took an antimalarial only were included in this series. Medical students and patients with incomplete records or under antibiotics were excluded. All students were outpatients.

Malaria parasitaemia was confirmed with thick blood smears stained Giemsa and examined for trophozoites of* Plasmodium falciparum.*

The following information was collected and analyzed: (1) age and gender and (2) nature, dosage, and duration of antimalarial treatment.

Statistical analysis was performed using the statistics software SPSS for windows (15.0 SPSS, Chicago). Data are represented as means ± SD when the distribution was normal and median with range when the distribution was not normal. Frequencies of different parameters of self-medication are expressed as proportions (%).

## 3. Results

During the study period, 458 students had been admitted in our centre and 133 (29.0%) had a diagnostic of malaria infection. The median age of the patients was 25.4 years (range: from 18 to 36 years). Self-medication practice was reported by 28 (21.1%) students. The majority of them were male (67.9%).

All the medicines taken were registered in DRC. All patients took an underdosage with these antimalarials. The duration of the treatment is not available in all of cases.

Self-medication was associated with the ingestion of artemisinin-based combination treatments (ACTs) in 14 cases (50%), sulfadoxine/pyrimethamine (SP) in 9 cases (32.1%), and quinine in 5 cases (19.9%). No case of ingestion of artesunate/artemether in monotherapy was found.

## 4. Discussion

To our knowledge, the present study is the first attempt to examine the notion of self-medication with antimalarials drugs in a university of Central Africa. In Kinshasa, malaria is highly endemic, stable with a perennial transmission. Malaria is primarily due to* Plasmodium falciparum* [5]. University of Kinshasa is located in one of four districts of Kinshasa, District of Mont Amba in the west region of this urban area. Prevalence of* Anopheles gambiense* was higher than that in other districts of the town [6–9].

In this report, 20% of students were examined in CSU after self-medication with antimalarials. This high figure shows the existence of a gap in the health care system including the freedom to buy drugs without prescription in pharmacies.

The analysis of dosage showed that self-prescribed antimalarial was very irrational. The duration of the treatment is not available in all cases. Similar observation was previously reported in Ivory Coast [10].

In this series, self-medication with SP was found in 32.1% of students. Recent study in DRC reported parasite genotyping which showed high frequencies of* dihydrofolate reductase (dhfr)* and* dihydropteroate synthase (dhps)* molecular SP-resistance markers, with 57% of the samples showing more than three mutations linked to SP resistance [11].

Self-medication with quinine was found in 20% of students. In national health care policy, quinine is reserved for severe malaria. This rate is high for a molecule reserved for severe disease in Congolese health care policy in management of malaria. A recent study supports our concerns and shows the appearance of an intermediate level of resistant forms to amino alcohols in the city of Kinshasa compared with other areas of Africa [11].

In the DRC, recent studies showed that ACTs were efficacious and safe. However, with an important self-medication, surveillance of efficacy of these drugs becomes a necessity since here there are recent concerns that the efficacy of such therapies has declined on the Thai-Cambodian border [12]. In our series, 50% of students used ACTs in self-medication. In comparison with our general population, fever treatment with ACTs is found with a frequency of 5% [13]. This difference can be due to better education and information in academic community.

Another crucial health problem is counterfeit antimalarials, mainly ACT and sulfadoxine-pyrimethamine in developing countries as Democratic Republic of Congo [14]. This situation increases the risk of artemisinin and SP resistance developed by the use of subtherapeutic dosage of these drugs [14].

Self-medication remains an important public health problem in high school students of Kinshasa. The recommendations of the National Program against malaria are not sufficiently known in these communities. This should draw the attention of all partners involved in the fight against malaria in DRC. The academic community should be a priority target for the information campaign about the disease and the dangers of self-medication and risk-taking counterfeit drugs. Health education on the appropriate use of antimalarials drugs is highly recommended. Training and information actions must be reinforced for a better care of malaria and to preserve efficacy and safety of ACTs and quinine in DRC.

## Additional Points

The small sample size is very unlikely to be representative of the broader population limiting the potential to make policy conclusions. The sample size is very small and unlikely to be representative as it only comes from one health facility. In addition, students may have misreported their self-medication.
